# Fallout from European Fires: An Estimate of Premature Deaths Attributable to Vegetation Smoke

**DOI:** 10.1289/ehp.125-A24

**Published:** 2017-01-01

**Authors:** Nancy Averett

**Affiliations:** Nancy Averett writes about science and the environment from Cincinnati, OH. Her work has been published in *Pacific Standard*, *Audubon*, *Discover*, *E/The Environmental Magazine*, and a variety of other publications.

Smoke from burning landscapes—including wildfires, prescribed burns, and agricultural clearing—can raise concentrations of fine particulate matter (PM_2.5_) and other harmful air pollutants in areas hundreds or even thousands of miles from the epicenter of a blaze.^1^ ^,^ ^2^ In a paper in this issue of *EHP*, researchers assessed landscape fires that occurred in Europe in 2005 and 2008, and estimated that the smoke they produced may have caused more than 2,500 premature deaths across more than two dozen countries.^3^

**Figure:**
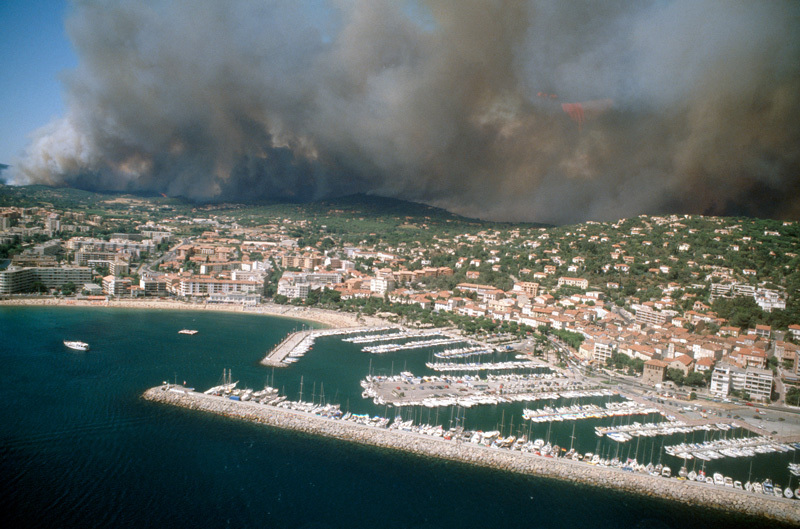
Southern France has an above-average number of wildfires, such as this 2003 blaze outside St. Tropez. PM_2.5_ pollution from fires like these can travel long distances to locales where fires themselves are less common. © STOCKFOLIO^®^/Alamy Stock Photo

“This paper focused on mortality effects [of smoke from landscape fires], but that’s just the tip of the iceberg,” says coauthor Timo Lanki, chief researcher at the National Institute for Health and Welfare in Kuopio, Finland. “There are so many more kinds of health effects that can result. … This is an indication of the scale of the problem, but only an indication.”

Lanki and colleagues combined satellite observations of radiant heat released by the fires and an atmospheric transport model of the smoke emissions that took into account humidity, weather, temperature, pressure, cloud cover, and solar radiation. They used these data to estimate daily concentrations of PM_2.5_ originating from fires, which they plotted across a 50×50-km^2^ grid spanning 27 European countries. Then they overlaid the grid with estimates of population and premature mortality.^3^

Sarah Henderson, an environmental epidemiologist who studies wildfire smoke and health at the British Columbia Centre for Disease Control, says she was impressed with the robustness of the researchers’ methodology. Henderson, who was not involved in the study, adds, “It’s interesting because mortality is not the same across geographic areas. In some countries, it could be higher because that population is more susceptible or more at risk for a variety of different factors that are challenging to capture. That they managed to build all of that into this study is pretty cool.”

Some regions of the world are especially heavily impacted by landscape fires. For instance, an average of 406,776 hectares (more than 1 million acres) burned each year across Portugal, Spain, Italy, Greece, and southern France between 2000 and 2013.^4^ Yet Lanki and his coauthors noted that deterioration of air quality due to landscape fires is not just a local problem; favorable atmospheric conditions can carry such pollutants thousands of miles.^1^ ^,^ ^2^ “Of course, if you are next to the fire, you will probably think of the health risks,” Lanki says, “but if you live further away you won’t necessarily.”

Most of the estimated premature deaths attributed to smoke exposure by the authors’ models would have resulted from relatively small increases in PM_2.5_—increments of less than 2 µg/m^3^. Henderson would like to have seen more discussion about the implications of that low number. “We haven’t really designed studies to test whether that small change in daily air quality does have a mortality impact,” she says. “We certainly don’t have the evidence to say that it doesn’t, but we also don’t necessarily have the evidence to say that it does.”

Overall, though, Henderson says the study offers vital information. “Smoke [exposure] from wildfires has not been considered as an important facet of these natural disasters. It’s really been considered a side product,” she says. “It’s true that lives and assets are at risk when these fires happen, but the bigger impacts are probably due to the smoke in the long run, because large populations are exposed.”

Henderson coauthored a 2012 paper that estimated 339,000 premature deaths occur around the world each year as a result of exposure to PM_2.5_ from landscape fires.^5^ A recent review of this and other papers concluded there is consistent evidence linking landscape fire smoke to respiratory disease in general, and especially to exacerbation of asthma and chronic obstructive pulmonary disease.^6^

In many countries people periodically burn vegetation for cropland management, and the authors noted that curbing this practice is one avenue policy makers could pursue to lower mortality from landscape fire smoke. They also suggested that officials protect public health by providing timely warnings and advice about using indoor air filters and staying indoors with the windows closed, as well as by raising awareness of the health effects of smoke and the need to prevent accidental fires.

As far as climate changes goes, Lanki says it doesn’t automatically mean there will be more wildfires. However, it does mean that in many regions there will be more often and longer periods of high fire risk, during which extra caution is needed.

## References

[1] Niemi JV Long-range transport episodes of fine particles in southern Finland during 1999–2007. Atmos Environ 43 6 1255 1264 2009, doi:10.1016/j.atmosenv.2008.11.022

[2] Sapkota A Impact of the 2002 Canadian forest fires on particulate matter air quality in Baltimore city. Environ Sci Technol 39 1 24 32 2005, doi:10.1021/es035311z 15667071

[3] Kollanus Mortality due to vegetation-fire originated PM_2.5_ exposure in Europe: assessment for the years 2005 and 2008. Environ Health Perspect 125 1 30 37 2017, doi:10.1289/EHP194 27472655 PMC5226696

[4] EFFIS Total Burnt Areas [spreadsheet].. http://forest.jrc.ec.europa.eu/media/cms_page_media/71/burnt_area_1.csv.

[5] Johnston FH Estimated global mortality attributable to smoke from landscape fires. Environ Health Perspect 120 5 695 701 2012, doi:10.1289/ehp.1104422 22456494 PMC3346787

[6] Reid CE Critical review of health impacts of wildfire smoke exposure. Environ Health Perspect 124 9 1334 1343 2016, doi:10.1289/ehp.1409277 27082891 PMC5010409

